# Side chain requirements for affinity and specificity in D5, an HIV-1 antibody derived from the V_H_1-69 germline segment

**DOI:** 10.1186/1471-2091-14-9

**Published:** 2013-04-08

**Authors:** Alex Stewart, Joseph S Harrison, Lauren K Regula, Jonathan R Lai

**Affiliations:** 1 Department of Biochemistry, Albert Einstein College of Medicine, 1300 Morris Park Avenue, Bronx, NY 10461, USA

## Abstract

**Background:**

Analysis of factors contributing to high affinity antibody-protein interactions provides insight into natural antibody evolution, and guides the design of antibodies with new or enhanced function. We previously studied the interaction between antibody D5 and its target, a designed protein based on HIV-1 gp41 known as 5-Helix, as a model system [Da Silva, G. F.; Harrison, J. S.; Lai, J. R., Biochemistry, 2010, 49, 5464–5472]. Antibody D5 represents an interesting case study because it is derived from the V_H_1-69 germline segment; this germline segment is characterized by a hydrophobic second heavy chain complementarity determining region (HCDR2) that constitutes the major functional paratope in D5 and several antibodies derived from the same progenitor.

**Results:**

Here we explore side chain requirements for affinity and specificity in D5 using phage display. Two D5-based libraries were prepared that contained diversity in all three light chain complementarity determining regions (LCDRs 1–3), and in the third HCDR (HCDR3). The first library allowed residues to vary among a restricted set of six amino acids (Tyr/Ala/Asp/Ser/His/Pro; D5-Lib-I). The second library was designed based on a survey of existing V_H_1-69 antibody structures (D5-Lib-II). Both libraries were subjected to multiple rounds of selection against 5-Helix, and individual clones characterized. We found that selectants from D5-Lib-I generally had moderate affinity and specificity, while many clones from D5-Lib-II exhibited D5-like properties. Additional analysis of the D5-Lib-II functional population revealed position-specific biases for particular amino acids, many that differed from the identity of those side chains in D5.

**Conclusions:**

Together these results suggest that there is some permissiveness for alternative side chains in the LCDRs and HCDR3 of D5, but that replacement with a minimal set of residues is not tolerated in this scaffold for 5-Helix recognition. This work provides novel information about this high-affinity interaction involving an antibody from the V_H_1-69 germline segment.

## Background

**Figure 1:**
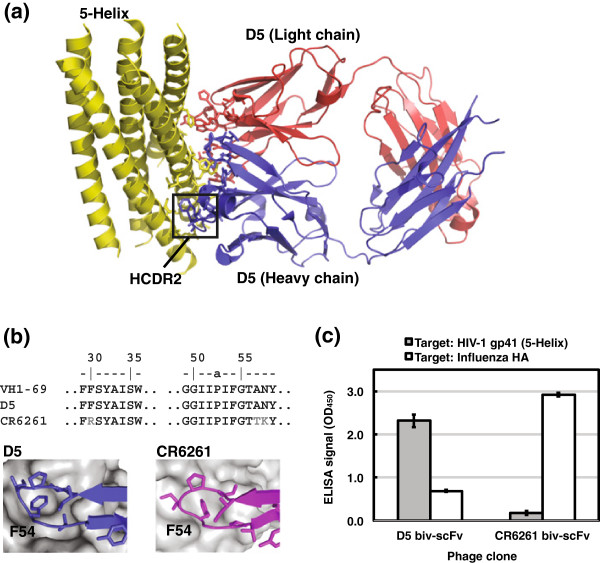
**Structure of the D5**-**5**-**Helix interaction and similarities to CR6261.** (**a**) Crystal structure of the D5-5-Helix complex reported by Luftig et al. (ref. 6, PDB ID 2CMR). The D5 light chain is colored in red, the D5 heavy chain in blue, and 5-Helix in yellow; side chains of residues involved in the interface are shown in stick. The critical HCDR2 is boxed. (**b**) Similarity of interactions involving HCDR2 in D5 and CR6261 (ref. 11, PDB ID 3GBN). In both antibody-antigen complexes, F54 of HCDR2 (a germline-encoded residue) inserts into a hydrophobic cleft on the antigen (here, shown in gray). The amino acid sequence of HCDR1 and HCDR2 regions from V_H_1-69, D5, and CR6261 are also shown. (**c**) Cross-reactivity analysis of phage clones displaying the bivalent scFv (biv-scFv) of D5 and CR6261. Phage titers were ~ 10^12^ infectious units/mL for both clones.

Specific and high affinity antibody-antigen interactions are critical to humoral immunity. Understanding antibody-antigen structure-function relationships provides basic information about molecular recognition and can aid in development of new research and therapeutic reagents [1-4]. We previously studied the interaction between the HIV-1 antibody D5 and its target (a protein mimic of HIV-1 gp41 known as ‘5-Helix’) as a model system for antibody-protein recognition (Figure 1a) [5-7]. This interaction has several unique characteristics. D5 has very high affinity for 5-Helix despite the fact that it was not evolved against this target (i.e., D5 was obtained from a ‘naïve’ phage antibody library) and the heavy and light chains are not heavily mutated relative to germline sequences [6,7]. The reported K_D_ values of D5 range from 50 pM to 20 nM, depending on the measurement technique (surface plasmon resonance, SPR, vs. isothermal titration calorimetry, ITC) and on the fragment (single-chain variable fragment, scFv, vs. antigen binding fragment, Fab, vs. IgG) [6-9]. In general, antibodies that bind proteins with high affinity contain extensively mutated (i.e., evolved) complementarity determining regions (CDRs); therefore, the lower mutation rate of D5 suggests that some naïve antibodies may have properties of evolved antibodies. Formation of the D5-5-Helix interface results in burial of > 1000 Å^2^ of combining site surface and residues in all six CDRs are involved in direct contacts with 5-Helix [6]. Most other antibody-antigen interactions are dominated by residues in heavy chain CDRs (HCDRs). Finally, the D5 heavy chain is derived from the V_H_1-69 germline segment and the HCDR1 and HCDR2 regions are identical to the germline. A striking similarity exists between the HCDR2-dominated interactions of D5 and those of another V_H_1-69 antibody, CR6261, which targets influenza HA (Figure 1b) [6,10-15]. The HCDR2 sequence and backbone conformations are highly similar, and in both cases the critical feature of the recognition involves insertion of F54 (a germline-encoded HCDR2 residue) into a hydrophobic cleft on the antigen [6,11]. Interestingly, while the HCDR1 regions are highly similar between both antibodies, an S30R mutation in CR6261 was shown to be a specificity determinant in its interaction with HA [14]. These results suggest that, while the hydrophobic HCDR2 may serve as a critical anchor point to engage in antigen recognition, other regions could play an important role in specificity determination. We previously reported that light chain contacts in D5 play an important role in affinity for 5-Helix [5].

A growing body of work has deciphered the rules for molecular recognition by antibodies and other immunoglobulin-like scaffolds. Recent efforts have focused on developing libraries containing restricted diversity segments within the CDRs of stable heavy and light chain variable domain (V_H_ and V_L_, respectively) frameworks [16-21]. This diversity is encoded by designed, synthetic oligonucleotides (‘synthetic antibodies’) which, when used in combination with screening by a display method (e.g., phage display, yeast display, or mRNA display), allows for identification of antibodies or antibody fragments with specificities and affinities comparable to or better than antibodies obtained from natural sources [22-26]. Additionally, restricted diversity libraries permit high-throughput mutagenesis studies of combining site residues to determine which characteristics most accurately reflect the physicochemical attributes of functional antibodies [4,16-18]. As an example, libraries in which residues at the CDRs are allowed to vary among subsets of amino acids (in some cases as few as two – Tyr and Ser) yield high affinity and specific binders in the context of regular immunoglobulin scaffolds and single-domain variants [4,16]. These results highlight the versatility of the immunoglobulin scaffold for molecular recognition.

Here we examine the factors that contribute to affinity and specificity of D5 by phage display using 5-Helix as a model antigen. The germline-encoded HCDR2 is believed to represent a critical feature of V_H_1-69 antibody recognition, as reflected in the apparent similarities in HCDR2 interactions between D5, CR6261, and others [6,10,14,15]. Therefore, we created two D5-based phage display libraries, in which the HCDR3 and the light chain (LCDRs) were allowed to vary using two different randomization schemes. We evaluated the abilities of these two libraries to specifically recognize 5-Helix with high affinity. This study provide insights into aspects of antibody recognition by the V_H_1-69 germline.

## Results

### Specificity Profiles of D5 and CR6261

Given the similarity of HCDR1 and HCDR2 among D5, CR6261 and the common V_H_1-69 germline segment [6,11-15], we sought to explore the degree of specificity of these two antibodies toward their native antigens. We expressed the single chain variable fragments (scFv) for both D5 and CR6261 in bivalent format on the surface of M13 bacteriophage as a fusion to the major coat protein pIII. Binding was tested against both 5-Helix and the CR6261 target HA. As shown in Figure 1c, both antibodies displayed high specificity toward their native antigens.

### Library design

We wondered to what degree the specificity and affinity in D5 was governed by CDRs other than HCDR1 and HCDR2 (LCDRs 1–3, and HCDR3). To explore this question, we designed and produced two synthetic antibody libraries based on D5 (these libraries are shown in Table 1). In Library I (D5-Lib-I), we introduced variation such that surface-exposed LCDR positions and residues in HCDR3 were permitted to vary in hexanomial fashion among Ala, Asp, Ser, Tyr, His and Pro (the ‘*BMT*’ codon was used where *B* = *C*/*G*/*T*, *M* = *A*/*C*). Synthetic antibody libraries containing binomial (Tyr/Ser) or tetranomial (Ala/Asp/Tyr/Ser) codon sets have been successful against many antigens in the context of other germline scaffolds [16-19,26]. The hexanomial scheme explored here also includes the positively-charged His and the conformationally-restricted Pro.

**Table 1:** Library design

	**Amino acid substitutions**^**A**^					
**Library**	**LCDR1**^**B**^ (**D5 WT permitted**)	**LCDR2**	**LCDR3**	**HCDR3**^**B**^ (**D5 WT permitted**)	**Theoretical diversity**	**Actual diversity**
D5-Lib-I	A/D/H/P/S/Y	A/D/H/P/S/Y	A/D/H/P/S/Y	A/D/H/P/S/Y	8 x 10^17^	3 x 10^9^
D5-Lib-II	Tailored^C^	Tailored^C^	Tailored^C^	A/C/D/E/G/K/N/R/S/T/Y/W	8 x 10^19^	3 x 10^9^

^A^ Amino substitutions encoded by limited diversity codon sets. For LCDRs 1 – 3: *BMT* (D5-Lib-I), or tailored codon sets listed in Table 2 (D5-Lib-II); for HCDR3 in all libraries: *DVK*. Nucleotide degeneracies: *M* = *A*/*C*, *B* = *C*/*G*/*T*, *D* = *A*/*G*/*T*, *V* = *A*/*C*/*G*, *K* = *G*/*T*.

^B^ In LCDR1 and HCDR3, the library synthesis was performed in such a manner to allow the D5 WT sequence as an alternative to the library sequences.

^C^ Tailored diversity codon sets used as shown in Table 2.

In the second library (Library II, D5-Lib-II), variation in the LCDRs was designed to mimic diversity of natural antibodies derived from the V_H_1-69 germline and paired with V_Κ_ light chains. We queried the PDB to identify antibodies with high homology to the V_H_1-69 germline segment that fulfilled three criteria: (1) their three-dimensional structures had been solved in complex with the antigen; (2) the antibody represented a product or variant of natural rearrangement (i.e., antibodies resulting from synthetic repertoires were not considered); (3) the sequences were unique. We compiled sequences from 24 total antibodies and found that 18 of these contained V_K_ light chains (see Additional file 1: Table S1). These antibodies target a variety of antigens (including small molecules, peptides, and proteins), and were isolated from phage display and other sources. In general, the LCDR loop lengths among these antibodies were similar to those found in D5. We examined each of the crystal structures and assessed LCDR positions for their importance in the structural paratope as gauged by surface area buried upon complex formation (Table 2). We assigned a qualitative ‘contact score’ (low, mid, or high) at each position based on the extent to which the residue at that position participated in structural paratopes across the datasets. In general, those positions with ‘high’ contact score contained side chains in which > 80% of the surface area was buried upon binding in three or more complexes. We determined the amino acid distribution at each position and designed restricted diversity codons to allow composition that reflected the distribution at each position or, in some cases, residues that had similar physicochemical properties to the natural distribution. At several positions, we allowed greater diversity than was observed in the structural dataset. For HCDR3, we allowed variation among the 12 residues encoded by the *DVK* codon, since HCDR3 has a high degree of variability among all antibody scaffolds [27].

**Table 2:** Library design

**D5 position**	**Contact score**^**A**^	**Amino acid distribution** (**frequency**)^**B**^	**Designed codon**^**C**^	**Encoded residues** (**coverage**^**D**^)
***LCDR1***				
E27	Low	E(3)/Q(13)/S(2)	Not varied	E (17%)
G28	Mid	D*(5)/G(2)/N(1)/Q(1)/S(8)/Y*(1)	*RRC*	D/N/G/S (88%)
Y30	Mid	G(3)/R(1)/S*(10)/Y*(2)/V(1)	*NDT*	C/D/F/G/H/I/N/L/R/S/V/Y (100%)
H31	Mid	H(4)/N*(5)/S(7)/T(2)	*NDT*	C/D/F/G/H/I/N/L/R/S/V/Y (61%)
W32	High	A(2)/D(1)/F(1)/G(1)/M(1)/N(2)/S(1)/W*(2)/Y*(6)/- (1)	*NDT*	C/D/F/G/H/I/N/L/R/S/V/Y (56%)
***LCDR2***				
Y49	High	Y*(18)	*TMT*	S/Y (100%)
K50	High	A(2)/D(1)/G(5)/K*(3)/L(2)/S(2)/Y*(3)	*HRC*	C/H/N/R/S/Y (28%)
S52	Low	S(17)/T(1)	*TMT*	S/Y (94%)
S53	Mid	F*(2)/N(3)/R*(3)/S(6)/T(3)/Y(1)	*HRC*	C/H/N/R/S/Y (73%)
A55	Low	A(9)/F(1)/H(3)/K(1)/P(1)/Q(1)/Y(2)	Not varied	A (50%)
***LCDR3***				
Y91	High	F(1)/G(3)/H(2)/R*(1)/S(3)/W*(2)/Y*(6)	*HRC*	C/H/N/R/S/Y (67%)
S92	High	A(1)/D*(1)/G(4)/L(1)/N*(3)/S*(4)/T(1)/W(1)/Y(2)	*KMT*	A/D/S/Y (44%)
N93	High	A(1)/D(1)/G(4)/H(1)/N*(5)/Q(1)/S*(1)/T(4)	*RVC*	A/D/G/N/S/T (89%)
Y94	Mid	L(3)/N(2)/S*(5)/T(3)/V(1)/W(2)/Y*(1)/- (1)	*DMT*	A/D/N/S/T/Y (61%)
P95	Mid	L(2)/P*(16)	Not varied	P (89%)
L96	High	F*(2)/I(1)/L*(1)/P(5)/R(1)/S(1)/W(4)/Y(3)	*YDT*	C/F/H/L/R/Y (39%)
T97	Low	R(1)/T(17)	Not varied	T (94%)

^A^ A ‘contact score’ for each position was assigned based on inspection of the antibody-antigen crystal structures in Additional file 1: Table S1. The involvement of each residue side chain was scored based on the buried surface area upon complex formation; positions that constituted a major element of the structural paratope in multiple antibodies were ranked ‘high’.

^B^ The amino acid identities and their observed frequency are listed. Residues that were heavily involved in the interaction in at least one antibody-antigen complex are indicated with an asterix. In some cases, loops lengths were shorter than D5; these are indicated by a ‘-‘at some positions.

^C^ Nucleotide degeneracies: *D* = *A*/*G*/*T*, *K* = *G*/*T*, *H* = *A*/*C*/*T*, *M* = *A*/*C*, *N* = *A*/*G*/*C*/*T*, *R* = *A*/*G*, *V* = *A*/*G*/*C*, *Y* = *C*/*T*.

^D^ ‘Coverage’ indicates the percentage of the naturally observed diversity that is encoded in the degenerate codon.

During synthesis of each library, we permitted ‘WT’ D5 side chain identity in both HCDR3 and LCDR1 by using template DNA that contained WT D5 side chain identity at these positions. Our rationale for this approach was to examine whether WT D5 sequences in HCDR3 and LCDR1 would be preferred to library sequences; if so, then clones containing these WT sequences should be selected over clones that contain library sequences. Both libraries were produced in bivalent scFv format with 3 x 10^9^ unique members each.

### Analysis of selectants

We screened both libraries for three rounds against 5-Helix. A large number of clones from the round 3 (R3) populations from both libraries were characterized by sequence analysis and monoclonal ELISA. Fifty-five of the 276 clones from D5-Lib-I R3 population contained library sequences and had positive but moderate binding signals for 5-Helix (OD_450_ > 0.4). Furthermore, these clones displayed moderate specificity for binding to 5-Helix (~4-fold ELISA signal for binding wells coated with 5-Helix in comparison to wells coated with BSA). In contrast, selection of D5-Lib-II resulted in a R3 population that was dominated by library members (186 of 192) that had strong positive ELISA signals for 5-Helix (OD_450_ > 1.0), and were highly specific (10-fold or higher over BSA). The fact that a high percentage of clones from the R3 population of D5-Lib-II contain library sequences and that many of these had strong, positive ELISA signals suggests that functional clones can be readily isolated from this library. In contrast, the lower amount of library sequences in R3 of D5-Lib-I and the generally modest binding signals from isolated clones indicate that functional clones are less readily selectable.

The sequences of functional clones from the D5-Lib-II selection were highly diverse (HCDR3 and LCDR1-3 sequences of 30 representative clones are shown in Table 3). Interestingly, most of the hits identified contained WT D5 HCDR3 region but incorporated library sequences in all three LCDRs. In contrast, the selectants from D5-Lib-I were divergent in HCDR3 although one clone, 6G12, contained the D5 HCDR3 segment (four representative clones are shown in Table 3). This observation suggests that solutions to high affinity 5-Helix recognition are restrictive in HCDR3 but permissive in the LCDRs. Furthermore, the high hit-rate obtained with D5-Lib-II is striking in light of the fact that it contains a 100-fold higher degree of theoretical diversity than does D5-Lib-I but was produced with an equivalent number of library members. This result suggests that the functional capacity for recognition in V_H_1-69 antibodies is enhanced with pairing of V_K_ domains containing appropriate amino acid substitutions. These findings are in agreement with our previous work demonstrating that extended interactions among the heavy and light chains are required for 5-Helix recognition by D5 [5].

**Table 3:** Sequences and phage ELISA profiles of selected clones

		**Amino acid sequence**^**A**^				**Phage ELISA signal ratio**^**B**^			
**Library**	**Clone**	**HCDR3**	**LCDR1**	**LCDR2**	**LCDR3**	**5**-**Helix**/**BSA** (**ratio**)	**5**-**Helix**/**LF** (**ratio**)	**5**-**Helix**/**KLH** (**ratio**)	**F**_***competitive***_^**C**^
	**D5 WT**^**D**^	**95 DNPTLL**	**28 GIYHW**	**49 YKASSL**	**91 YSNYPL**				
D5-Lib-I	6G10	**DAPPSP**	**SPDYY**	**DASYYH**	**DDHSPY**	0.4/0.1 (5)	0.4/0.2 (3)	0.4/0.1 (3)	1.0
	6G12	***DNPTLL***	***GIYHW***	**SDYYSY**	**HAA**Y**PH**	0.6/0.1 (6)	0.6/0.3 (2)	0.6/0.2 (3)	0.8
	6D9	**DYPHHY**	***GIYHW***	**YHDYYP**	**SYP**D**PH**	0.4/0.1 (5)	0.4/0.2 (3)	0.4/0.1 (3)	0.7
	6B11	**SHPDDD**	**DDADD**	**PYYASD**	**YDDHPP**	0.4/0.1 (5)	0.4/0.2 (2)	0.4/0.2 (2)	1.0
D5-Lib-II	25B8	***DNPTLL***	**NINRN**	**SRASRL**	**RYNYPY**	2.9/0.1 (22)	2.9/0.1 (21)	2.9/0.2 (12)	0.2
	25C10	***DNPTLL***	**NIYGN**	**SRASRL**	**RYTYPL**	3.2/0.1 (34)	3.2/0.1 (33)	3.2/0.2 (18)	0.1
	25D8	***DNPTLL***	**GISSN**	**SRAYRL**	**RATYPL**	3.2/0.1 (30)	3.2/0.1 (22)	3.2/0.2 (18)	0.2
	25A10	***DNPTLL***	**SISHN**	**SNASRL**	**YSNYPL**	3.2/0.1 (23)	3.2/0.1 (22)	3.2/0.2 (14)	0.1
	25D9	***DNPTLL***	**DILGR**	**YRASRL**	**RANYPL**	3.0/0.1 (23)	3.0/0.1 (24)	3.0/0.2 (17)	0.2
	25G8	***DNPTLL***	**SIGRS**	**YRASRL**	**SSTTPL**	2.1/0.1 (24)	2.1/0.1 (25)	2.1/0.1 (16)	0.1
	25C6	***DNPTLL***	**NISSR**	**SNAYHL**	**RSNYPH**	1.8/0.1 (25)	1.8/0.1 (20)	1.8/0.1 (15)	0.2
	25C12	***DNPTLL***	**DISSV**	**YNASRL**	**RANNPH**	2.2/0.1 (23)	2.2/0.1 (18)	2.2/0.2 (10)	0.3
	25D6	***DNPTLL***	**NIYSN**	**SSASRL**	**NSNNPH**	1.6/0.1 (21)	1.6/0.1 (19)	1.6/0.1 (14)	0.1
	25D3	***DNPTLL***	**NIHRR**	**YSAYSL**	**NSDYPH**	1.6/0.1 (20)	1.6/0.1 (17)	1.6/0.2 (10)	0.1
	25F1	***DNPTLL***	**GINNS**	**SRASRL**	**YSSTPH**	2.3/0.1 (24)	2.3/0.1 (20)	2.3/0.1 (15)	0.1
	25B6	***DNPTLL***	**NIRSG**	**YRASRL**	**YDDYPH**	1.4/0.1 (16)	1.4/0.1 (15)	1.4/0.1 (11)	0.1
	25F10	***DNPTLL***	**GIHNR**	**SHASNL**	**YSNYPL**	1.7/0.1 (22)	1.7/0.1 (22)	1.7/0.1 (13)	0.1
	25A12	***DNPTLL***	**GIHRY**	**YHASRL**	**RANYPY**	3.2/0.1 (27)	3.2/0.2 (20)	3.2/0.3 (12)	0.1
	25A5	***DNPTLL***	**SIRSH**	**SRASRL**	**SSDYPH**	3.5/0.1 (24)	3.5/0.1 (24)	3.5/0.2 (15)	0.1
	16F6	***DNPTLL***	**SISSI**	**SNASRL**	**RSNYPF**	3.0/0.2 (13)	3.0/0.3 (11)	3.0/0.7 (4)	0.8
	25C5	***DNPTLL***	**DIHDY**	**SRASRL**	**NDSYPY**	0.7/0.1 (10)	0.7/0.1 (8)	0.7/0.1 (6)	0.5
	16D10	***DNPTLL***	**NIRGS**	**YRASRL**	**HSDYPH**	3.0/0.1 (20)	3.0/0.2 (20)	3.0/0.5 (7)	0.7
	16G6	***DNPTLL***	**GIRRS**	**SNASHL**	**YYNYPR**	2.3/0.2 (14)	2.3/0.2 (12)	2.3/0.3 (7)	0.3
	16B7	***DNPTLL***	**NILRL**	**YYASSL**	**RADYPY**	3.0/0.1 (33)	3.0/0.1 (22)	3.0/0.5 (6)	0.6
	16E12	***DNPTLL***	**GIRRS**	**SNASHL**	**YYNYPR**	3.1/0.4 (9)	3.1/0.3 (12)	3.1/0.7 (5)	0.1
	25F12	***DNPTLL***	**GIIGH**	**YNASRL**	**RDDYPL**	2.7/0.1 (27)	2.7/0.1 (23)	2.7/0.2 (12)	0.2
	25B4	***DNPTLL***	**GIRNG**	**SRASHL**	**YYAYPH**	2.9/0.1 (29)	2.9/0.1 (31)	2.9/0.1 (22)	0.1
	16E8	***DNPTLL***	**SIYGS**	**SHASHL**	**NDTYPH**	3.0/0.1 (21)	3.0/0.2 (12)	3.0/0.3 (9)	0.4
	25E1	***DNPTLL***	**DIGGS**	**SHAYSL**	**HATYPH**	3.0/0.1 (32)	3.0/0.1 (28)	3.0/0.2 (20)	0.1
	25A6	***DNPTLL***	**GIYDG**	**SRAYYL**	**RANYPH**	3.1/0.2 (20)	3.1/0.1 (25)	3.1/0.2 (16)	0.1
	16E3	***DNPTLL***	**GIRYG**	**SRASRL**	**HYAYPH**	3.0/0.3 (11)	3.0/0.4 (8)	3.0/0.6 (5)	0.7
	2H10	***DNPTLL***	**DIYRS**	**SRASRL**	**YSNYPL**	1.9/0.1 (15)	1.9/0.1 (19)	1.9/0.2 (10)	0.1
	25C4	***DNPTLL***	**SILGS**	**YRASHL**	**YSNYPL**	2.5/0.1 (32)	2.5/0.1 (26)	2.5/0.1 (19)	0.1
	16F5	***DNPTLL***	**NINDH**	**SRAYRL**	**YADTPF**	3.0/0.2 (19)	3.0/0.2 (12)	3.0/0.3 (11)	0.2

^A^ In cases where selection of the entire CDR was observed (for HCDR3 and LCDR3), the sequence is italicized. Positions that were not randomized are underlined.

^B^ For each clone, the raw ELISA signal (OD_450_) is shown against both 5-Helix and control proteins BSA, lactoferrin (LF), or keyhole limpet hemocyanin (KLH). The ratio of ELISA signals for 5-Helix over each of the three control proteins is shown in parentheses.

^C^ The reduction in ELISA signal observed upon preincubation with free 5-Helix (500 nM, D5-Lib-I; 40 nM, D5-Lib-II) as a fraction of the signal observed in the absence of the competitor.

^D^ Kabat numbering for the first residue of each CDR is shown for D5 WT.

We used high-throughput ELISAs to assess specificity and affinity among the selectants. To examine specificity, we performed the phage ELISA against 5-Helix and two control proteins in addition to BSA: lactoferrin (LF) and keyhole limpet hemocyanin (KLH). LF is a ubiquitous protein found in many tissues, but was not introduced in the selection (BSA was used as a blocking reagent) and therefore provided a good control for testing specificity against unrelated proteins. KLH is known to be strongly immunogenic and is frequently employed as a carrier protein for immunogenicity and vaccination studies [28]. We surmised that polyspecific clones (i.e., those displaying properties of unevolved antibodies) would have reactivity with this protein; therefore cross-reactivity with KLH served as another stringent measure of specificity. By determining the ratio of ELISA reactivity for 5-Helix over BSA, LF, or KLH we could rapidly assess the specificity of each selectant in a high-throughput manner.

In addition, we performed a single-point competitive phage ELISA experiment in which each phage clone was preincubated with soluble 5-Helix prior to capture in an ELISA well containing immobilized 5-Helix. Those clones with higher affinity should therefore have a higher occupancy of 5-Helix in the combining site from the preincubation, hence a lower ELISA signal. Similar strategies have been used to assess other synthetic antibody libraries. In general, phage clones in which the ELISA signal is reduced by > 50% upon preincubation of 10 nM or 100 nM free antigen results in antibodies with low or mid nanomolar dissociation constants (respectively) when the corresponding protein Fabs were purified and assayed by SPR [17,27]. We found that preincubation of D5-Lib-II selectants with 40 nM free 5-Helix provided a large dynamic range of ELISA signals among selectants, therefore we used this concentration to assess relative affinities for these clones. Selectants from D5-Lib-I were generally lower affinity and consequently necessitated a higher concentration of free 5-Helix (500 nM) for the competition assay. The data are represented as the fraction of ELISA signal observed in the presence of the free 5-Helix relative to the signal observed without competitor (F_competitive_, Table 3).

Table 3 lists representative clones from D5-Lib-I and D5-Lib-II selection along with results from specificity profile analysis and single-point competition ELISA. This analysis revealed that selectants from D5-Lib-II contained varying levels of specificity for 5-Helix over BSA, LF, and KLH although generally the selectivity for 5-Helix was strong. The ratio of ELISA signals for 5-Helix over each of the control protein was at least 5-fold in all cases and, for most clones, an over 10-fold ratio was observed against all three control proteins. Furthermore, the affinity, as assessed by F_competitive_, was high in most cases since the 40 nM free 5-Helix resulted in more than 50% reduction in ELISA signal (F_competitive_ < 0.5) for nearly all of the clones. Notably, three of the clones with the best selectivity and affinity profiles (25A10, 2H10, and 25C4) contained LCDR3 sequences that are identical to WT D5. However, similarity to the D5 LCDR3 region was not an absolute necessity; clone 25D6 exhibited high affinity and specificity but contained no homology to D5 in the LCDR3 region.

Selectants from D5-Lib-I were generally less specific and had poor affinity. The ratio of ELISA signals for 5-Helix over BSA did not exceed 6-fold. Furthermore, only moderate competition was observed upon addition of 500 nM free 5-Helix in two cases (6G12 and 6D9). In the other two cases, no competition was observed. The results obtained with D5-Lib-I and D5-Lib-II suggest that restricted diversity in the context of this interaction is insufficient to provide highly functional clones, despite the fact that sequence space in D5-Lib-I is much more adequately sampled than in D5-Lib-II.

### Conformational specificity

Antibody D5 inhibits HIV-1 infection by binding the N- and C-heptad repeat regions of gp41 (NHR and CHR, respectively) and sequestering a conformation known as the ‘extended intermediate’ in the gp41-mediate viral membrane fusion pathway that is required for virus entry [6,29,30]. The target for D5, 5-Helix, is an engineered protein containing the NHR and CHR segments designed to mimic the ‘extended intermediate’ [29,31,32]. The critical HCDR2 loop of D5 projects into a hydrophobic cleft that should only be present in this conformational form of gp41 [6]. Therefore, antibody D5 is predicted to exhibit conformational specificity for the gp41 NHR and CHR – the antibody should bind mimics of the extended intermediate but not the ‘post-fusion’ form of this proteins (a six-helix bundle) [31,32].

**Figure 2:**
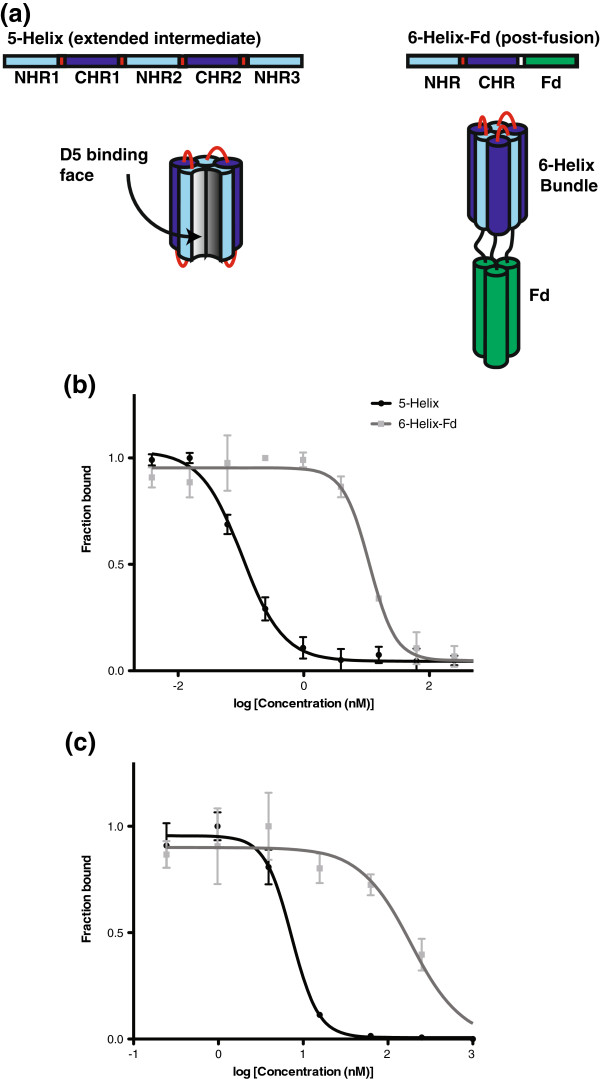
**5**-**Helix and 6**-**Helix**-**Fd design and competitive ELISA.** Design of 5-Helix and 6-Helix-Fd (**a**), and competitive ELISA with D5 scFv (**b**, phage-bound bivalent scFv; **c**, purified monovalent scFv).

We sought to define the conformational preferences of D5 and the selectants from D5-Lib-II. We prepared a designed protein containing the gp41 NHR and CHR segments which mimics the six-helix bundle ‘post-fusion’ conformation (‘6-Helix-Fd’) [31,32]. This protein consists of the NHR linked to the CHR by a short linker, followed by a trimeric coiled-coil segment from T4 fibritin (Foldon, Fd) to promote trimerization (Figure 2a) [33]. 6-Helix-Fd was purified from *E*. *coli* by standard procedures and found to be α-helical by circular dichroism consistent with design (see Additional file 1: Table S1). To explore conformational specificity of the antibody clones, we performed competitive ELISA assays in which binding to immobilized 5-Helix was inhibited by binding free 5-Helix or free 6-Helix-Fd (sample data for D5 are shown in Figure 2b). The IC_50_ obtained by competition with free 5-Helix provides an estimate for binding activity. Furthermore, the relative IC_50_ obtained by competition with 6-Helix-Fd enables evaluation of preference for the extended intermediate conformation over the post-fusion conformation. These results are summarized in Table 4 (full plots can be found in the Additional file 1: Table S1).

**Table 4:** **Apparent affinities of clones from D5**-**Lib**-**II selection against 5**-**Helix**

	**On**-**phage** (**bivalent**)		**Off**-**phage** (**monovalent**)	
**Clone**	**IC**_**50 **_**for 5**-**Helix**^**A**^/**nM**	**IC**_**50 **_**for 6**-**Helix**-**Fd**^**A**^/**nM**	**IC**_**50 **_**for 5**-**Helix**^**A**^/**nM**	**IC**_**50 **_**for 6**-**Helix**-**Fd**^**A**^/**nM**
D5 WT	0.1 (0.07-0.1)	11 (8–15)	7.3 (5.3-10)	290
25B8	3.1 (1.8-5.3)	~100	ND^B^	ND^B^
25C10	1.8 (1.3-2.7)	28 (21–37)	56 (40–79)	*NC*^C^
25D8	5.8 (4.2-7.5)	~3000	ND^B^	ND^B^
25A10	1.6 (1.3-2.0)	260 (150–450)	5.4 (3.8-7.7)	~500
25D9	1.7 (1.4-2.1)	230 (150–360)	ND^B^	ND^B^
25G8	7.2 (4.2-12.3)	300 (120–750)	ND^B^	ND^B^
25C6	32 (24–44)	~1000	ND^B^	ND^B^
25C12	100 (31–300)	~1000	ND^B^	ND^B^
25D6	0.5 (0.3-0.8)	~80	ND^B^	ND^B^
25D3	17 (9.9-30)	170 (70–390)	ND^B^	ND^B^
25F1	0.9 (0.7-1.2)	40 (20–79)	ND^B^	ND^B^
25B6	0.3 (0.2-0.4)	23 (15–36)	0.6 (0.3-1.0)	94 (56–160)
25F10	0.2 (0.2-0.3)	22 (16–30)	ND^B^	ND^B^
25A12	2.2 (1.2-4.0)	220 (70–670)	34 (23–52)	280 (120–640)

^A^ 95% Confidence intervals from data fitting are shown in parentheses.

^B^ ND, not determined.

^C^*NC*, no convergence observed upon data fitting, suggesting weak or insignificant binding.

We previously reported an IC_50_ of D5 for 5-Helix of 0.1 nM, and here we determined an IC_50_ for 6-Helix-Fd of 11 nM (concentrations calculated for the trimer; Figure 2b) [19]. Therefore, the D5 is able to discriminate the extended and post-fusion conformations of gp41 by 100-fold difference in apparent affinity. Selectants from D5-Lib-II ranged in their apparent affinity for 5-Helix, some were similar to D5 (e.g., 25D6, 25B6, and 25F10) but others had 10- or 100-fold higher IC_50_ (e.g., 25C10 and 25G8, respectively). However, most retained their ability to distinguish 6-Helix-Fd from 5-Helix by ~100-fold difference in apparent affinity. In one case, 25D8, specificity for 5-Helix over 6-Helix-Fd was enhanced relative to D5 (~500-fold selectivity). We have previously shown that analysis of binding to 5-Helix in this format, with the antibody fragment displayed on phage, agrees well with results using the purified antibody fragment [19]. To further validate this assumption, we purified the scFv for D5 and several of the clones for binding analysis. In general, the IC_50_ obtained for the purified scFv proteins were ~10-fold higher than those observed on-phage. However, the overall trends were consistent with results on-phage for the clones examined.

### Positional preferences

Diverse populations of phage selectants can be used to assess positional requirements for protein-protein interactions by determining the degree of conservation for a particular residue in a functional selection (here, 5-Helix binding) relative to a selection for protein display [5,34-36]. In some cases, these datasets have been used to infer energetic consequences of mutation provided certain assumptions are validated [5,34-36]. We performed a selection of D5-Lib-II against the anti-FLAG antibody M2 to obtain a reference dataset to quantify display biases. A FLAG epitope sequence was included at the N-terminus of our scFv construct; therefore selection against M2 should provide readout of display bias. We compiled sequences for 179 clones from the 5-Helix selection that scored well in terms of specificity profile analysis (OD_450_ ratios of four-fold or higher for 5-Helix over each of the controls). For the reference (display) set, we compiled 168 sequences that had a strong, positive ELISA signal for M2 binding. At each position, we determined the percentage occurrence of each residue and ranked from 1st to 4th most frequent from the functional selection. At positions 49 and 52 of LCDR2, the randomization encoded variation between just two amino acids (Tyr and Ser). These data are represented in Table 5, with the identity of the WT D5 residue preceding the residue number in the first column and the four most frequent residues from the functional selection listed in order of frequency. In cases where additional residues were permitted and observed, these were binned together into a fifth class, ‘other’. For each residue, we calculated the ratio between occurrence in the functional and display selections (F/D); this analysis provide a direct evaluation of the extent to which a particular side chain is enriched in the functional (5-Helix) selection population over the display (anti-FLAG) selection. Stronger preferences for function are indicated by both high occurrence (% of population in the functional selection) and F/D > 1. While this analysis provides a rough guideline for identifying biases for recognition, caution must be used in analysis of these data since there is no error estimate associated with occurrence or F/D.

**Table 5:** **Apparent affinities of clones from D5**-**Lib**-**II Selection against 5**-**Helix**

	**1st**^**A**^				**2nd**^**A**^				**3rd**^**A**^				**4th**^**A**^				**Others**^**A**^		
		**Function % (5-Helix)**	**Display % (Anti-FLAG)**	**Function/Display**^C^		**Function % (5-Helix)**	**Display % (Anti-FLAG)**	**Function/Display**^C^		**Function % (5-Helix)**	**Display % (Anti-FLAG)**	**Function/Display**^C^		**Function % (5-Helix)**	**Display % (Anti-FLAG)**	**Function/Display**^C^	**Function % (5-Helix)**	**Display % (Anti-FLAG)**	**Function/Display**^C^
**G28**	***G***^***B***^	***38***	***34***	**1**.**1**	**N**	**25**	**21**	**1**.**2**	**D**	**19**	**20**	**1**.**0**	**S**	**19**	**25**	**0**.**8**	**0**	**0**	-
Y30*	**H**	**22**	**9**	**2**.**4**	**S**	**13**	**4**	**3**.**2**	**R**	**13**	**11**	**1**.**2**	**G**	**11**	**9**	**1**.**2**	**41**	**67**	**0**.**6**
**H31**	**R**	**23**	**5**	**4**.**6**	**S**	**19**	**4**	**4**.**8**	**G**	**15**	**5**	**3**.**0**	**N**	**14**	**16**	**0**.**9**	**29**	**70**	**0**.**4**
**W32**	**S**	**26**	**14**	**1**.**9**	**N**	**19**	**7**	**2**.**7**	**R**	**16**	**8**	**2**.**0**	***H***	***14***	***11***	***1***.***3***	**25**	**60**	**0**.**4**
**Y49**	**S**	**60**	**45**	**1**.**3**	***Y***	***40***	***55***	***0***.***7***		-	-	-		-	-	-	-	-	-
K50*	***R***	***61***	***10***	***6***.***1***	**N**	**15**	**18**	**0**.**8**	**H**	**13**	**23**	**0**.**6**	**S**	**7**	**17**	**0**.**4**	**4**	**32**	**0**.**1**
**S52**	***S***^***B***^	***77***	***49***	***1***.***6***	**Y**	**23**	**51**	**0**.**4**		-	-	-		-	-	-	-	-	-
**S53**	**R**	**54**	**19**	**2**.**8**	**H**	**22**	**17**	**1**.**3**	***S***^***B***^	***11***	***22***	***0***.***5***	**N**	**8**	**16**	**0**.**5**	**5**	**26**	**0**.**2**
**Y91**	**R**	**36**	**13**	**2**.**8**	**N**	**20**	**17**	**1**.**2**	**S**	**18**	**15**	**1**.**2**	***Y***^***B***^	***14***	***24***	**0**.**6**	**12**	**31**	**0**.**4**
**S92**	***S***^***B***^	***34***	***30***	***1***.***1***	**A**	**27**	**20**	**1**.**4**	**Y**	**27**	**30**	**0**.**9**	**D**	**12**	**20**	**0**.**6**	**0**	**0**	-
**N93**	***N***^***B***^	***31***	***28***	***1***.***1***	**D**	**18**	**10**	**1**.**8**	**A**	**17**	**13**	**1**.**3**	**T**	**17**	**28**	**0**.**6**	**17**	**21**	**0**.**8**
Y94*	***Y***^***B***^	***71***	***20***	***3***.***6***	**T**	**13**	**21**	**0**.**6**	**S**	**6**	**17**	**0**.**4**	**N**	**5**	**11**	**0**.**4**	**5**	**31**	**0**.**2**
**L96**	**H**	**35**	**14**	**2**.**5**	**F**	**19**	**15**	**1**.**3**	**Y**	**19**	**19**	**1**.**0**	***L***^***B***^	***15***	***25***	***0***.***6***	**12**	**27**	**0**.**4**

^A^ For each position, the percentage of the population of analyzed clones is shown for the 1st, 2nd, 3rd, and 4th most frequently observed residue from the 5-Helix selection. The WT D5 residue is indicated prior to the position number in the first column and the “hotspot” positions identified by Da Silva et al. are marked by asterisk. If two of the amino acids exhibit the same frequency, the one with the higher Function/Display ratio^C^ will rank in a higher order. In cases where additional substitutions were permitted and observed, these were binned into a 5th category labeled ‘other’. At positions 49 and 52, only two residues (Tyr and Ser) were permitted. The data shown here were compiled from 178 sequences from the 5-Helix selection and 169 sequences from the display selection.

^B^ Amino acids shown in the four most frequently observed at each position among library members that are identical to WT D5 are shown in gray italic font. For positions that the WT residue was not encoded in the library due to codon degeneracy, amino acids that have the closest physiochemical properties to the WT that is among the four most frequent residues are in black italic fonts.

^C^ Ratio of percent frequency observed from functional (5-Helix) selection and percent frequency observed from display (anti-FLAG) selection.

Nearly every position in the LCDRs exhibits specific preferences in the population for functional selection, as indicated by F/D > 1 for the 1st and 2nd most frequently observed residue. Four positions correspond to residues in D5 that had high energetic cost for mutation to alanine (ΔΔG_Ala-WT_ ≥ 1.0 kcal/mol, ‘hot spot’ residues) in our previous scanning mutagenesis experiments: Y30, K50, Y94, and L96 (marked with an asterix in Table 5). All of these positions had a preference for the most commonly-observed residue from the D5-Lib-II selection (F/D >1). Polar and charged residues were preferred at LCDR positions 30 and 32, despite the fact that these positions are occupied by large hydrophobes in D5 (Tyr and Trp, respectively). We previously demonstrated that Y30 has ΔΔG_Ala-WT_ of 1.0 kcal/mol [5]. Therefore, variations in other portions of the LCDRs must allow for less hydrophobic residues at position 30. In positions 31 (LCDR1), 49 and 53 (LCDR2), the preferred residues (Arg, Ser, Arg, respectively) were not the D5 WT residue (His, Tyr, Ser, respectively), despite the fact that the WT residue was included in the randomization set. In contrast, in positions 92, 93, and 94 of LCDR3, the WT D5 side chain identity was preferred. This result suggests that LCDR3 diversity is more restrictive. Tyr was highly favored in position 94 (F/D = 3.6); this position lies at the center of the interface and corresponds to a strong hot spot residue in D5 (Y30 has ΔΔG_Ala-WT_ of 2.6 kcal/mol). Position 50 in LCDR2, which corresponds to another strong hot spot residue in D5 (K50, ΔΔG_Ala-WT_ = 2.1 kcal/mol) [5], had a strong preference for cationic side chains. Arg and His accounted for > 70% of the population; and Arg had a F/D of 6.1. In position 96, His was preferred but this position is occupied by Leu in D5 and is another hot spot residue (ΔΔG_Ala-WT_ = 1.5 kcal/mol).

Overall, the population analysis of functionally-selected R3 clones suggest that there is some degree of flexibility and permissiveness for 5-Helix recognition by D5, but that LCDR3 positions 92, 93, and 94 favor the WT D5 residues. It is somewhat surprising that hydrophobic residues, particularly Tyr, were not more strongly favored at the LCDR positions in the functional selection. Tyr is the most commonly observed residue in functional and naïve CDR positions and plays critical roles in recognition by natural and synthetic antibodies [4,16]. In four of the 13 positions examined, Tyr is found at the corresponding site in D5 (Y30, Y49, Y91, and Y94); furthermore, Tyr was permitted at these positions and seven others in D5-Lib-II but was only strongly favored at position 94. In contrast, cationic or polar residues were abundant in most positions. These results suggest that LCDR contacts in this context provide polar or ionic contributions to binding, either directly or indirectly. Position 94, which showed the highest degree of preference for Tyr, was also the residue found to have the highest ΔΔG_Ala-WT_ in our previous alanine scanning studies. Examination of the clones in Table 3, however, demonstrates that Tyr at this position is not an absolute requirement – clones 25D6 and 25F1 rival D5 in terms of specificity and affinity yet contain polar residues at position 94 (Asn and Thr, respectively). However, both of these clones contained Tyr at other LCDR positions.

Another interesting observation is that restrictiveness in positional side chain identity for D5-Lib-II selectants against 5-Helix did not correlate with ΔΔG_Ala-WT_ values previously observed in D5. For example, Y30 and L96 of D5 were found to have ΔΔG_Ala-WT_ ≥ 1.0 kcal/mol in the alanine scanning studies but these positions had only moderate functional preferences, and these preferences were not for the WT D5 side chain identities even though Tyr and Leu were encoded in the randomization set at positions 30 and 96. These results match comprehensive scanning studies on the human growth hormone-receptor interaction in which ‘hot spot’ residues (i.e., those with ΔΔG_Ala-WT_ ≥ 1.0 kcal/mol) correlated with some, but not all, positions that had stringent requirements for side chain identity [37]. Furthermore, the preferred amino acids in the LCDR positions did not correlate with those most frequently observed in the analysis of the 18 V_H_1-69-related antibodies; and those positions that had the most stringent amino acid preferences were not necessarily those assigned a high contact score in the structural analysis. Therefore, the functional preferences for LCDR side chain identity are likely context-dependent.

**Figure 3:**
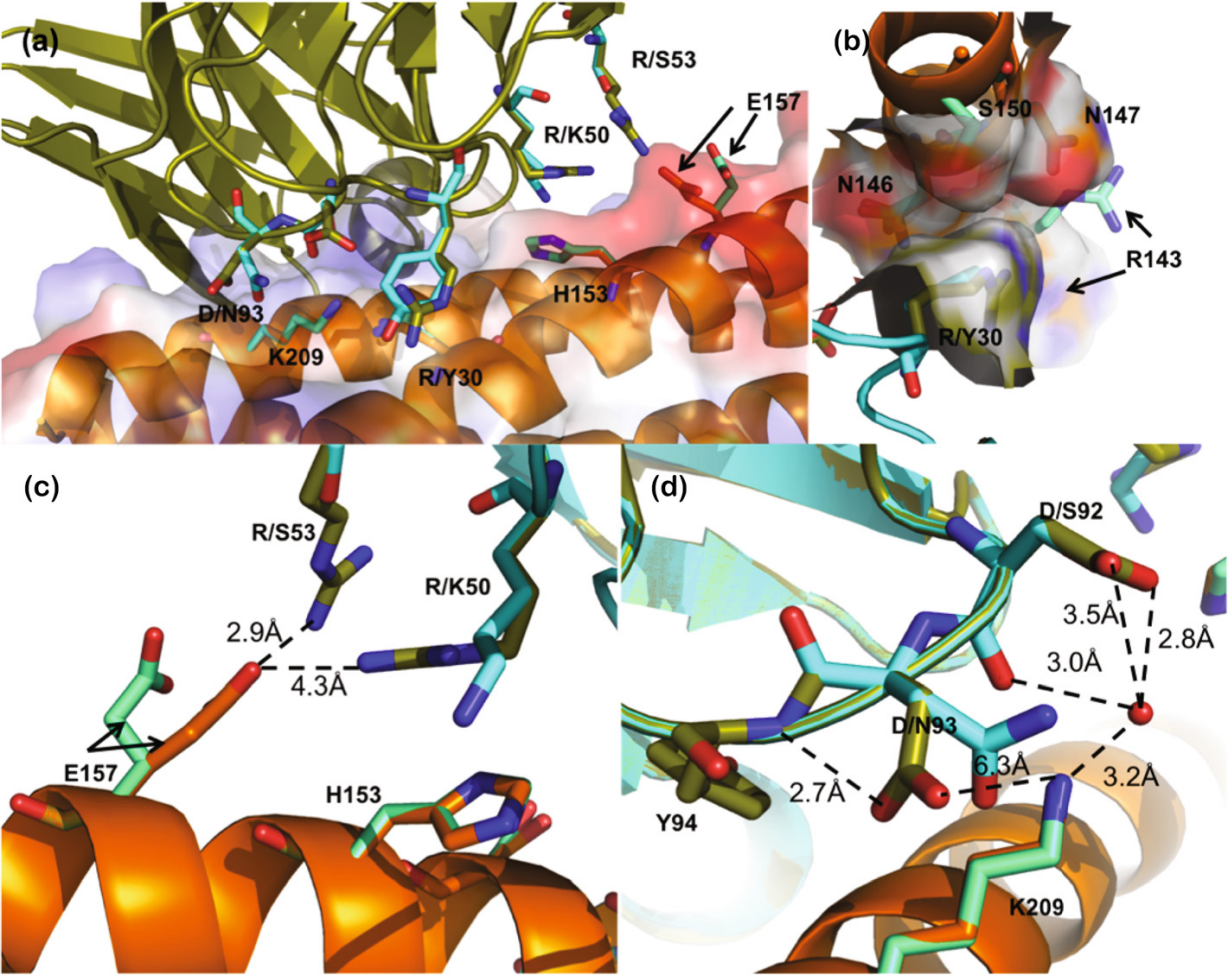
**Optimized complementarity in the 25B6**-**5Helix interaction revealed by modeling.** (**a**) Structural model of 25B6 complexed with 5-Helix (in cartoon) is superimposed onto the crystal structure of D5-5-Helix complex (PDB ID 2CMR). 25B6 is colored in deep olive, D5 in cyan, and 5-Helix in orange. Side chains of residues participating in the interaction are shown as stick; for 5-Helix, the side chains are colored teal in the D5-5-Helix model, and orange in the 25B6-5-Helix model. Electrostatic potential surface of 5-Helix from the 25B6 modeling is shown as well. (**b**) Potential of R30 in 25B6 to contact several residues on the periphery of the 5-Helix. (**c**) Potential interaction of E157 (5-Helix) with R50 and R53 of 25B6. (**d**) Potential polar interactions between 25B6 and 5-Helix mediated through D92 and D93 and a water molecule.

Among the analyzed clones, the combining site of 25B6 maximizes both hydrophobic and electrostatic features given in the D5-Lib-II diversity (Table 3). By our metrics, 25B6 scFv has a higher relative affinity compared to D5 (IC_50_ of 0.6 nM for 25B6 and 7.3 nM for D5). This clone contains positive charges in positions 30, 50, and 53 (Arg), and negative charges at positions 92 and 93 (Asp). Overall, Asp was not a frequent substitute in this selection; however, Asp at positions 92 and 93 may enhance interaction with the positively charge residues in the N-terminal heptad repeat (NHR) [5]. To better understand the nature of potential charged residue interactions at those positions, we used the FixedBBProteinDesign module in Rosetta3 to obtain a model of the 25B6 interaction with 5-Helix [38,39]. The crystal structure of the D5-5-Helix (PDB ID 2CMR) and structural model of 25B6 are superimposed in Figure 3. All three Arg residues in 25B6 have the potential to engage in favorable electrostatic interactions with 5-Helix. In position 30, the long carbon chain of Arg in 25B6 acts as the edge of an overall concave surface into which the α-helices of 5-Helix are nestled. This predicted interaction is similar to that of Y30 in D5 [5,6]. Similarly, the extended length of Arg in position 50 and 53 results in the potential for formation of electrostatic interactions with E156 of the CHR of the 5-Helix. The long carbon chain of R50 can potentially make van der Waals contact with H153. On the other hand, the two Asp residues that occupy position 92 and 93 can form salt bridges with, or provide electrostatic complementarity to K574 of 5-Helix. Such interactions may contribute to the high affinity interaction between 25B6 and 5-Helix.

## Discussion

Our high throughput analysis of selectants from D5-Lib-II indicates that the pool contained diverse clones with a variety of binding affinities. Interestingly, most clones maintained their specificity at both the antigen level (as judged by the high throughput ELISA ‘specificity analysis’) and many retained conformational specificity (as judged by recognition for 5-Helix over 6-Helix-Fd). Global sequence analysis of functional clones suggested LCDR1 and LCDR2 could accommodate many residues while LCDR3 was more restrictive. This may reflect biases of natural antibodies to utilize LCDR3 as a predominant contact region. Furthermore, we previously reported that the D5 LCDR3 contains several hot spot residues [5]. Therefore, it seems this region is important for recognition of 5-Helix in multiple contexts. On a clonal level, it appears there are many recognition solutions while retaining D5-like affinity and specificity. As an example, clones 25D6, 25F1, 25B6, and 25F10 were comparable to D5 by our metrics but had very different LCDR features. In particular, 25B6 contains Arg in position 30, 50, and 53, and Asp in position 92 and 93. It is conceivable for the charged residues in the light chain enhance stability and solubility on a very hydrophobic V_H_ antigen-binding surface; it is also reasonable to speculate that the charge residues can be used to improve overall binding interface by electrostatic complementarity.

The observation that D5-Lib-I did not yield D5-like clones is surprising in light of the fact that the critical HCDR2 loop of the V_H_1-69 germline segment is included in these two repertoires. Interactions of two hydrophobic residues (I53 and F54) in the HCDR2 of CR6261 were enough to trigger B cell activation [14]. And importantly, a handful of somatic hypermutations were enough to allow D5 to bind 5-Helix in low nanomolar to high picomolar affinity. Thus, inclusion of residues that have important physiochemical properties biased toward protein-protein interaction should be sufficient to yield functional clones. However, our results indicated that interactions with 5-Helix using a V_H_1-69 germline clearly require extended interactions of a very specific nature involving the light chain [5]. Libraries based on the V_H_1-69 scaffold may therefore require a much larger diversity to achieve high affinity and specificity. We conclude that while there are some requirements in side chains of the LCDR positions (as demonstrated by the moderate functionality of clones from D5-Lib-I), there is some permissiveness for affinity and specificity of the 5-Helix antibody recognition provided the correct attributes are present.

Humoral immunity requires a delicate balance of a broadly reactive naïve repertoire (i.e., ‘germline-encoded’ antibodies) and highly specific evolved antibodies. Structural and biochemical work on hapten-binding antibodies has demonstrated that germline-encoded antibodies typically exhibit polyreactivity through dynamic CDRs [39-41]. Mutations that arise during affinity maturation reduce the flexibility of the CDR segments such that they are locked into a conformation that is productive for antigen binding. This “conformation locking” mechanism may have played a role in dominance of WT HCDR3 because of the degeneracy of the codon set did not allow Pro to be permitted in position 97 in D5-Lib-II, a residue that is important for the interaction with D5.

However, it is less obvious how protein-binding antibodies evolve specificity and affinity. Studies with an anti-hen egg white lysozyme (HEL) antibody and its germline-encoded progenitors suggests that affinity maturation in this case involves optimization of CDR loop conformations by mutation of a residue at the V_H_-V_L_ interface [42]. Similar to other protein-protein interactions, the affinity of protein-antibody interactions is significantly influenced by the complementarity of the two interacting surfaces and the exclusion of water at the intermolecular interface [43]. In the case of the anti-HEL antibodies, a key mutation at the V_H_-V_L_ interface resulted in HCDR1 and HCDR2 displacements that optimized the overall antigen-binding surface. This model is unlikely to be generalizable since the vast majority of matured protein-antibody interactions involve a high degree of mutation in the CDR segments. Furthermore, in vitro evolution of protein-binding antibodies can be achieved by mutagenesis of the CDR segments alone [44].

We previously examined the D5-5-Helix interaction by scanning mutagenesis and found that the high affinity results from extended interactions involving the V_H_ and V_L_. Here we find that both affinity and specificity can be altered with mutations in the LCDRs and HCDR3. The fact that positions in the functional paratope of the D5-5-Helix complex (as determined by a large ΔΔG_Ala-WT_ in alanine scanning mutagenesis studies) were permissive while retaining affinity and specificity suggests that there are multiple solutions to evolution of binding. However, the hexanomial restricted diversity library D5-Lib-I did not yield high affinity clones; this result suggests that some functional constraints do exist, and that these constraints differ from other germline scaffolds.

## Conclusions

Here we have explored side chain requirements for binding and specificity in D5, a model HIV-1 antibody derived from the V_H_1-69 germline segment. These results provide a template for future synthetic antibody libraries based on this germline scaffold, and provide novel insights into protein-antibody recognition.

## Methods

### Expression and purification of 5-Helix and 6-Helix-Fd

5-Helix was isolated essentially as described [5,29]. A synthetic gene encoding the 6-Helix-Fd sequence (see Additional file 1: Table S1 for details) was obtained from a commercial supplier (Genewiz, South Plainfield, NJ) and cloned into pET22b using NdeI and XhoI restriction sites to produce the expression plasmid pLR22. E. coli BL21(DE3) cells (Invitrogen, Madison, WI) harboring pLR22 were grown in LB broth at 37°C to OD_600_ ~0.6, and expression induced by the addition of 0.5 mM isopropyl-β-D-thiogalactopyranose (IPTG). The culture was incubated overnight at 15°C. The cells were isolated by centrifugation and lysed in a French pressure cell. The soluble and insoluble fractions were separated by ultracentrifugation; the 6-Helix-Fd protein was contained in the insoluble fraction. The insoluble fraction was resuspended in 6 M GdnHCl, the cell debris removed by centrifugation, and the supernatant applied directly to Ni-NTA resin (Qiagen, Valencia, CA). The resin was washed with 20 mL of 6 M GdnHCl/20 mM imidazole, then with 20 mL of 6 M GdnHCl/50 mM imidazole and the protein was eluted with several fractions 6 M GdnHCl/200-500 nM imidazole. The fractions containing the purified protein were pooled, and refolded by dialysis into phosphate-buffered saline (PBS, pH 7). The protein was either used immediately for analysis or flash frozen and stored at – 80°C.

### Phage display

The D5 scFv display phagemid pJH3 [5] was altered to allow bivalent D5 scFv display to produce phagemid pJH3B. The open reading frame (ORF) consisting of the D5 scFv sequence upstream of the C-terminal 188 residues of M13 phage coat protein pIII (pIII-CT) in pJH3 was expanded to include an IgG hinge region and a GCN4 leucine zipper segment between the scFv and pIII-CT. The final construct (pJH3B) has an ORF containing the OmpA periplasmic export sequence, an N-terminal FLAG epitope (for detection), the D5 scFv, the IgG hinge region, GCN4, and pIII-CT as a single chimeric fusion protein. Phage ELISA and Western blotting confirmed functional display of the bivalent D5 scFv assembly on phage particles (not shown). Bivalent display of the CR6261 scFv was similar; a synthetic DNA fragment encoding the CR6261 scFv codon optimized for E. coli was obtained from DNA 2.0 (Menlo Park, CA) for construction of this display vector. For cross-reactivity studies, influenza HA was purchased from Sino Biological Inc. (Beijing, P.R. China).

Phage growth and ELISA analysis was performed using standard methods [5,45]. *E*. *coli* XL1-Blue harboring the appropriate phagemid were grown to mid-log phase in LB broth supplemented with 5 μg/mL tetracycline and 50 μg/mL carbenicillin. Helper phage VCSM13 (Stratagene, Santa Clara, CA) or M13K07 (New England Biolabs, Ipswitch, MA) were added to 10^10^ plaque-forming units (pfu)/mL followed by 25 μg/mL kanamycin. The culture was grown 18 hrs at 30°C, the cells removed by centrifugation, and phage precipitated by addition of 3% (w/v) NaCl and 4% (w/v) PEG 8000. The phage were pelleted by centrifugation and resuspended in PBS containing 1% BSA. For phage ELISA, wells of Costar EIA/RIA high-binding plates were coated with antigen (typically 0.2 – 1.0 μg/well) in 100 mM NaHCO_3_ pH 8.5 at room temperature for 1 hr or at 4°C overnight. The well solutions were decanted and unbound sites were blocked by incubation with PBS containing 1% BSA for 1 hr. The wells were washed with PBS containing 0.05% Tween 20 (PBS-T), then the phage solutions were added and allowed to bind at room temperature for 0.5 – 1 hr. The phage solutions were decanted, the wells washed 5 – 7 times with PBS-T, then a solution containing anti-M13-horseradish peroxidase conjugate (GE Healthcare, Piscataway, NJ) was added and allowed to bind for 0.5 – 1 hr as directed by the manufacturer. The wells were washed with PBS-T and developed by addition of a 3, 3’, 5, 5’-Tetramethylbenzidine (TMB) substrate. The ELISA signal was quantified either by direct measurement of blue color absorbance (OD_650_) or by quenching with H_2_SO_4_ after 10 mins and determining the OD at 450 nm.

### Library construction

Library DNA was prepared using Kunkel mutagenesis [5,19,45]. A template clone based on pJH3B (see above) was prepared in which LCDR2 and LCDR3 regions were replaced with poly rare-Arg codon-containing segments. We have found that rare-Arg codon-containing segments provide enhanced selection relative to similar strategies that use stop codon-containing template clones because the residual rare Arg-codon template is less prone to growth advantages. Single-stranded, uridine-enriched DNA (ss-dU-DNA) of rare Arg-containing template clone was prepared in CJ2036 E. coli (NEB) using established protocols. Kunkel mutagenesis performed using 5’-phosphorylated primers corresponding to the reverse complement of the designed library sequences as previously described [5]. In general, Kunkel reactions contained 10 μg of template DNA, three-fold excess of library primer, three units of T7 polymerase and two units of T4 ligase. These reactions were incubated at room temperature overnight and then the library DNA purified using a QIAgen PCR purification kit.

The *E*. *coli* clone SS320 was used for library electroporations and was prepared by mating MC1016 and XL1-Blue [19,45]. The purified library DNA was electroporated into SS320 competent cells that had been preinfected with VCSM13 or K07. Typical electroporations were performed with 350 μL of competent cells and 10 μg of purified library DNA in 0.2 cm cuvettes using a BioRad Gene Pulser electroporator (2.5 kV and 200 Ω). Cells were allowed to recover for 45 min at 37°C and then large scale phage production was performed as above. Library phage were suspended in PBS and either used immediately for screening or stored at – 80°C. The final library phage preparations had high infectious titer (10^12^ – 10^13^ pfu/mL). The quality was assessed by large-scale DNA sequencing of phage clones; in all cases, the libraries were highly diverse in sequence and contained ~ 30% functional library members.

### Library selection and analysis

Library sorting was performed in Costar EIA/RIA plates; the antigen was immobilized into plate wells as above. Library phage were added and allowed to bind for 1 – 2 hrs, then the wells were washed extensively with PBS-T. The binding phage were eluted by treatment with 100 μL of 100 mM glycine HCl pH 2.0 for 10 min, and the solution was neutralized by addition of 50 μL of 2 M Tris, pH 8.0. The neutralized phage solution was then added to 5 mL of log-phase XL1-Blue *E*. *coli* in 2×YT broth supplemented with tetracycline. After 1 hr, 50 μg/mL carbencillin along with helper phage were added and the culture was grown at 37°C for 1 hr. Subsequently, 25 mL of 2×YT containing 50 μg/mL carbenicillin and 25 μg/mL kanamycin were added and the culture was grown at 30°C for 18 hrs. The cells were removed by centrifugation, then the phage was isolated as above and used immediately for subsequent rounds of infection. Selection progress was monitored by 1) large-scale sequencing of the phage populations (to look for enrichment of library clones) and 2) output phage titers from wells containing the target to wells containing a BSA control.

Individual clones were grown small scale for high-throughput phage ELISA analysis in deep 96-well plates. Cultures of 1 mL LB broth containing carbencillin were inoculated with colonies corresponding to selectants, helper phage were added (10^10^ pfu/mL) and the culture grown at 30°C for 18 hrs. The cells were removed by centrifugation and the supernatant applied directly to ELISA plate wells in which the antigen or control protein had been immobilized. Phage solutions were allowed to bind for 15 mins, the wells washed with PBS-T, and then the bound phage detected with the anti-M13/HRP conjugate as above. For specificity profile analysis, LF and KLH were purchased from Sigma-Aldrich (St. Louis, MO). Single-point competitive ELISAs were similar except that the phage solutions were preincubated with 40 nM 5-Helix for 30 min before addition to wells containing the immobilized 5-Helix. Both specificity profile analysis and single point competition analysis were spotchecked for reproducibility and, in general, gave consistent results among independent experiments. Competitive phage ELISAs were performed essentially as described [19].

### Expression of scFv proteins and monoclonal ELISAs

Phagemid vectors were converted to expression vectors by replacement of the hinge, GCN4 and pIII-CT segment downstream of the scFv segment with a hexahistidine tag. The scFv proteins were expressed in the periplasm of *E*. *coli* BL21. Cultures were grown in low-phosphate media at 30°C for 14 – 16 hrs and the cells harvested by centrifugation. Cell lysis was achieved by treatment with Bug Buster (Novagen, Madison, WI). The lysate was clarified by ultracentrifugation and purified by nickel affinity chromatography. Purified scFv proteins were dialyzed into PBS then used immediately for analysis or flash frozen and stored at – 80°C. Analysis by ELISA was similar to phage ELISA except that an anti-FLAG/HRP conjugate was used to detect the scFv protein (a FLAG epitope is present at the N-terminus).

### Structural modeling of 25B6

To model the 25B6-5-Helix interaction, we used the FixedBBProteinDesign module in Rosetta3 using the co-crystal structure of D5 and 5-Helix as a starting model (PDB ID 2CMR) [6,38,39]. Amino acid substitutions were incorporated in the light chain to match the 25B6 sequence; the lowest energy structure from 200 runs is represented in Figure 3. The following command line options used were used: minimize_sidechains, ex1, ex2, nstruct 200, use_input_sc, and linmem_ig 10.

## Authors’ contributions

AS, JSH, and JRL participated in the design of the study and in the draft of the manuscript. AS, JSH, and LKR carried out the experiments described in the text. All authors read and approved the manuscript.

## Supplementary Material

Additional file 1 Amino acid alignment of D5 and CR6261 variable domains; list of structures used for design of D5-Lib-II; design and CD characterization of 6-Helix-Fd; full competitive ELISA profiles. Click here for file
