# Supplementary material for: Side chain requirements for affinity and specificity in D5, an HIV-1 antibody derived from the VH1-69 germline segment
Source: BMC Biochem. 2013 Apr 8;14:9. doi: 10.1186/1471-2091-14-9 (PMC3626704; doi:10.1186/1471-2091-14-9)
Supplement: Additional file 1 — Amino acid alignment of D5 and CR6261 variable domains; list of structures used for design of D5-Lib-II; design and CD characterization of 6-Helix-Fd; full competitive ELISA profiles. [file 1471-2091-14-9-S1.pdf]

Supporting Information for: Side Chain Requirements for Affinity and Specificity in D5, an HIV-1 Antibody Derived from the V_H1-69 Germline Segment

Alex Stewart, Joseph S. Harrison, Lauren K. Regula, and Jonathan R. Lai*

Department of Biochemistry, Albert Einstein College of Medicine, 1300 Morris Park Avenue, Bronx, New York 10461

Sequence comparison of D5 and CR6261 V_L and V_H domains.

```
Light chain
D5-VL       diqmtqspst lsasigdrvt itcrasegiy hwlawyqqkp gkapklliyk asslasgaps
CR6261-VL   qsvltqppsv saapgqkvti scsgsssnig ndyvswyqql pgtapklliy dnnkrpsgip

D5-VL       rfsgsgsgtd ftltisslqp ddfatyycqq ysnypltfgg gtkleikrtv
CR6261-VL   drfsgsksgt satlgitglq tgdeanyyca twdrrptayv vfgggtkltv


Heavy chaina (CDR regions shown in gray)    HCDR1                       HCDR2
D5-VH       qvqlvqsgae vrkpgasvkv sckasgdtfs syaiswvrqa pgqglewmgg iipifgtany
CR6261-VH   evqlvesgae vkkpgssvkv sckasggpfr syaiswvrqa pgqgpewmgg iipifgttky
                                                   HCDR3
D5-VH       aqafqgrvti taneststay melsslrsed taiyycardn ptllgsdywg agtlvtvssa
CR6261-VH   apkfqgrvti taddfagtvy melsslrsed tamyycakhm gyqvretmdv wgkgttvtvs
```

Table S2 – Structures Used for Design of D5-Lib-II

Antibody	PDB ID (ref.)	Target
D5	2CMR (S1)	HIV-1 gp41 (5-Helix)
412D	2QAD (S2)	HIV-1 gp120
M75	2HKF (S3)	Human carbonic anhydrase IX
Ru5	1FE8 (S4)	Von Willebrand factor A3 domain
36-65	2A6D (S5)	Arsonate / peptide mimic
Unnamed	2ZJS (S6)	SecYE
Aqc2	1MHP (S7)	VLA1 I-domain
Unnamed	2B2X (S8)	VLA1 I-domain
23c3	3CXD (S9)	Osteopontin / peptide mimic
80r	2GHW (S10)	SARS S1 receptor binding domain
X5	2B4C (S11)	HIV-1 gp120
GC1008	3EO1 (S12)	Transforming growth factor β
4E10	2FX7 (S13)	HIV-1 gp41
17b	1GC1 (S14)	HIV-1 gp120
Unnamed	3G6J (S15)	C3b
R3Mab	3GRW (S16)	FGF receptor 3
Unnamed	2QQN (S17)	Neuropilin 1/2
E2	3BN9 (S18)	MT-SP1/matriptase

References

S1) Luftig MA, Mattu M, Di Giovine P, Geleziunas R, Hrin R, Barbato G, Bianchi E, Miller MD, Pessi A, Carfí A. Structural basis for HIV-1 neutralization by a gp41 fusion intermediate-directed antibody. Nat Struct Mol Biol. 2006 13(8):740-7.

S2) Huang CC, Lam SN, Acharya P, Tang M, Xiang SH, Hussan SS, Stanfield RL, Robinson J, Sodroski J, Wilson IA, Wyatt R, Bewley CA, Kwong PD. Structures of the CCR5 N terminus and of a tyrosine-sulfated antibody with HIV-1 gp120 and CD4. Science. 2007 317(5846):1930-4.

S3) Král V, Mader P, Collard R, Fábry M, Horejsí M, Rezácová P, Kozísek M, Závada J, Sedlácek J, Rulísek L, Brynda J. Stabilization of antibody structure upon association to a human carbonic anhydrase IX epitope studied by X-ray crystallography, microcalorimetry, and molecular dynamics simulations. Proteins. 2008 71(3):1275-87.

S4) Romijn RA, Bouma B, Wuyster W, Gros P, Kroon J, Sixma JJ, Huizinga EG. Identification of the collagen-binding site of the von Willebrand factor A3-domain. J Biol Chem. 2001 276(13):9985-91.

S5) Sethi DK, Agarwal A, Manivel V, Rao KV, Salunke DM. Differential epitope positioning within the germline antibody paratope enhances promiscuity in the primary immune response. Immunity. 2006 24(4):429-38.

S6) Tsukazaki T, Mori H, Fukai S, Ishitani R, Mori T, Dohmae N, Perederina A, Sugita Y, Vassylyev DG, Ito K, Nureki O. Conformational transition of Sec machinery inferred from bacterial SecYE structures. Nature. 2008 455(7215):988-91.

S7) Karpusas M, Ferrant J, Weinreb PH, Carmillo A, Taylor FR, Garber EA. Crystal structure of the alpha1beta1 integrin I domain in complex with an antibody Fab fragment. J Mol Biol. 2003 327(5):1031-41.
S8) Clark LA, Boriack-Sjodin PA, Eldredge J, Fitch C, Friedman B, Hanf KJ, Jarpe M, Liparoto SF, Li Y, Lugovskoy A, Miller S, Rushe M, Sherman W, Simon K, Van Vlijmen H. Affinity enhancement of an in vivo matured therapeutic antibody using structure-based computational design. Protein Sci. 2006 15(5):949-60.
S9) Du J, Hou S, Zhong C, Lai Z, Yang H, Dai J, Zhang D, Wang H, Guo Y, Ding J. Molecular basis of recognition of human osteopontin by 23C3, a potential therapeutic antibody for treatment of rheumatoid arthritis. J Mol Biol. 2008 382(4):835-42.
S10) Hwang WC, Lin Y, Santelli E, Sui J, Jaroszewski L, Stec B, Farzan M, Marasco WA, Liddington RC. Structural basis of neutralization by a human anti-severe acute respiratory syndrome spike protein antibody, 80R. J Biol Chem. 2006 281(45):34610-6.
S11) Huang CC, Tang M, Zhang MY, Majeed S, Montabana E, Stanfield RL, Dimitrov DS, Korber B, Sodroski J, Wilson IA, Wyatt R, Kwong PD. Structure of a V3-containing HIV-1 gp120 core. Science. 2005 310(5750):1025-8.
S12) Grütter C, Wilkinson T, Turner R, Podichetty S, Finch D, McCourt M, Loning S, Jermutus L, Grütter MG. A cytokine-neutralizing antibody as a structural mimetic of 2 receptor interactions. Proc Natl Acad Sci USA. 2008 105(51):20251-6.
S13) Cardoso RM, Brunel FM, Ferguson S, Zwick M, Burton DR, Dawson PE, Wilson IA. Structural basis of enhanced binding of extended and helically constrained peptide epitopes of the broadly neutralizing HIV-1 antibody 4E10. J Mol Biol. 2007 365(5):1533-44.
S14) Kwong PD, Wyatt R, Robinson J, Sweet RW, Sodroski J, Hendrickson WA. Structure of an HIV gp120 envelope glycoprotein in complex with the CD4 receptor and a neutralizing human antibody. Nature. 1998 393(6686):648-59.
S15) Katschke KJ Jr, Stawicki S, Yin J, Steffek M, Xi H, Sturgeon L, Hass PE, Loyet KM, Deforge L, Wu Y, van Lookeren Campagne M, Wiesmann C. Structural and functional analysis of a C3b-specific antibody that selectively inhibits the alternative pathway of complement. J Biol Chem. 2009 284(16):10473-9.
S16) Qing J, Du X, Chen Y, Chan P, Li H, Wu P, Marsters S, Stawicki S, Tien J, Totpal K, Ross S, Stinson S, Dornan D, French D, Wang QR, Stephan JP, Wu Y, Wiesmann C, Ashkenazi A. Antibody-based targeting of FGFR3 in bladder carcinoma and t(4;14)-positive multiple myeloma in mice. J Clin Invest. 2009 119(5):1216-29.
S17) Appleton BA, Wu P, Maloney J, Yin J, Liang WC, Stawicki S, Mortara K, Bowman KK, Elliott JM, Desmarais W, Bazan JF, Bagri A, Tessier-Lavigne M, Koch AW, Wu Y, Watts RJ, Wiesmann C. Structural studies of neuropilin/antibody complexes provide insights into semaphorin and VEGF binding. EMBO J. 200726(23):4902-12.
S18) Farady CJ, Egea PF, Schneider EL, Darragh MR, Craik CS. Structure of an Fab-protease complex reveals a highly specific non-canonical mechanism of inhibition. J Mol Biol. 2008 380(2):351-60.

Design and characterization of 6-Helix-Fd

Amino acid sequence of 6-Helix-Fd

```
|--------------CHR----------------|--Link--|------------------NHR------------
MWMEWDREINNYTSLIHSLIEESQNQQEKNEQELLGGKGGSSGIVQQQNNLLRAIEAQQHLLQLTVWGIKQLQARI


--|-Link|---------Fd--------------|
LGTGGSGGYIPEAPRDGQAYVRKDGEWVLLSTFLGENLYFQSHHHHHH
```

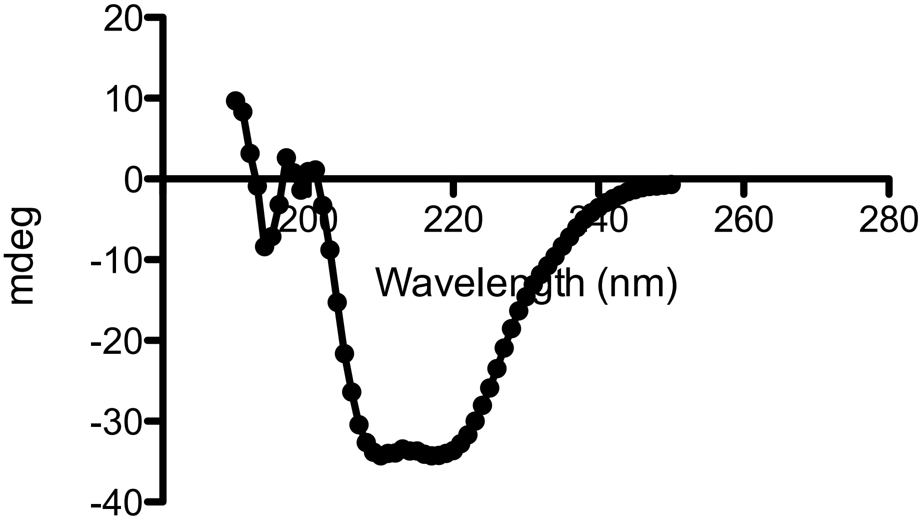

Figure S1 – Circular dichroism spectrum of 6-Helix-Fd.

Competitive ELISAs with 5-Helix and 6-Helix-Fd.

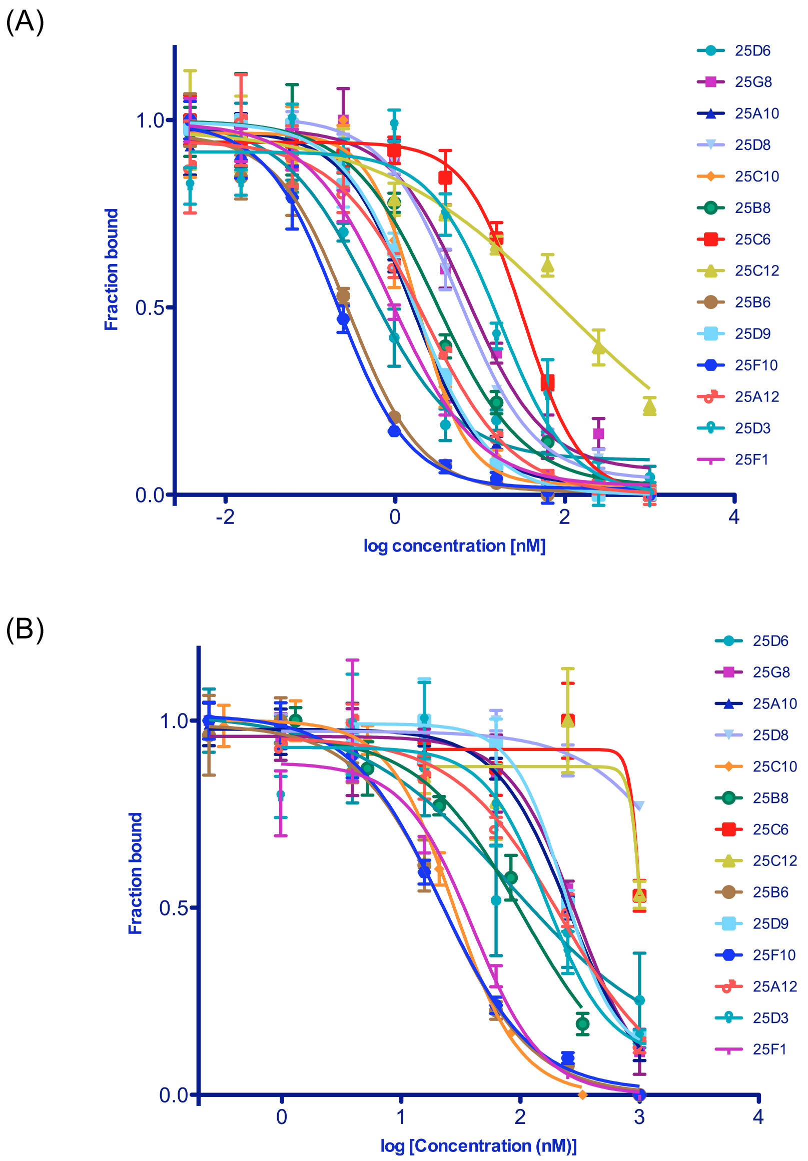

Figure S2 – Competitive ELISA for bivalent phage-displayed scFv fragments. Binding to immobilized 5-Helix was competed with free 5-Helix (A) or 6-Helix-Fd (B).

(A)

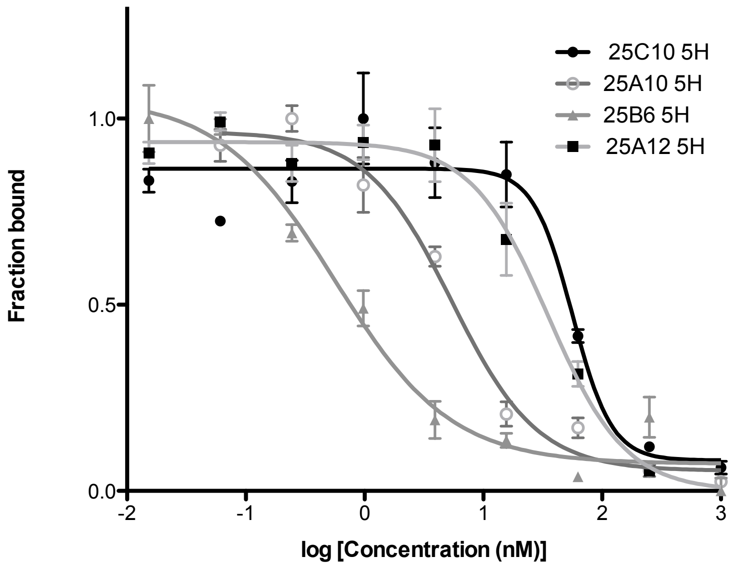

(B)

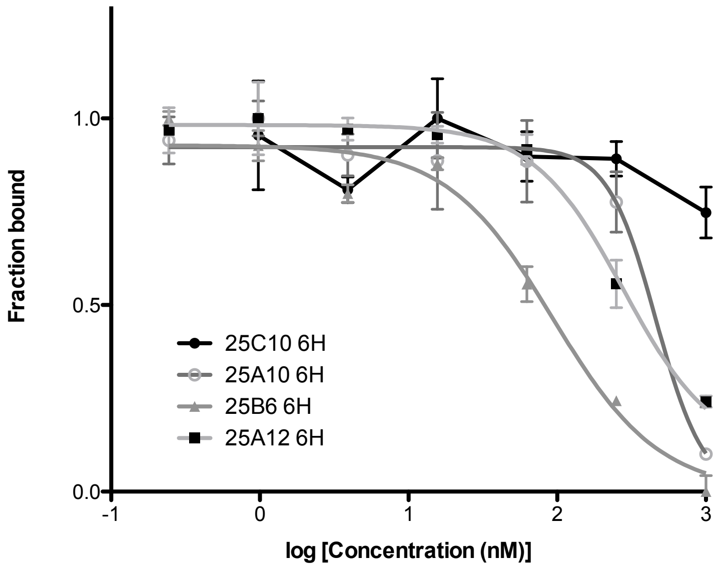

Figure S3 – Competitive ELISA for purified scFv fragments. Binding to immobilized 5-Helix was competed with free 5-Helix (A) or 6-Helix-Fd (B).
